# Public Policy by Syllogism? Does Logic Hold the Answer to Better Policy Outcomes?

**DOI:** 10.1177/0193841X251344054

**Published:** 2025-05-17

**Authors:** Joseph Drew, Rene Villano, Dana McQuestin, Masato Miyazaki

**Affiliations:** 1 College of Human and Social Futures, University of Newcastle, Callaghan, NSW, Australia; 2 1319 UNE Business School, University of New England, Armidale, Australia; 3 Department of Economics, Saitama University, Saitama, Japan

**Keywords:** public policymaking, policy success, logic, practical syllogism

## Abstract

Sometimes, public policy outcomes disappoint when unintended consequences arise. In many such cases, the problems might be traced back to poor reasoning. For most of antiquity, logic was considered the core element for successful human endeavour. In this work, we argue that Aristotelian logic – specifically, the syllogism – remains highly relevant and could offer significant benefits for the development of sound public policy. To demonstrate the value of logic for contemporary public policymaking, we first provide an accessible explanation of the practical syllogism. Following this we set out our method for testing the value of syllogistic reasoning against an example of real-world public policymaking. Thereafter, we test both the validity and truth of the apparent syllogism. We conclude that the use of a practical syllogism would have prevented unintended harm from arising in the instance under consideration and also offer our thoughts around generalisability and future research directions.

## Introduction

Public policy sometimes disappoints – either because the objectives are not met, or because unintended side-effects emerge (Sanderson, 2002; Weiss, 1990). Indeed, policy failure often features in media headlines and quite understandably generates resentment and frustration amongst citizens (Drew, 2025). Furthermore, there is large scholarly literature on policy failure as well as the grey areas in between (see Howlett (2012) for an excellent summary). This large literature on apparently avoidable policy failures has also elicited quite a deal of exasperation by scholars (see Tavares, 2018; Gendzwil et al., 2021).

A significant part of the problem revolves around the issue of timing – when public policy is being formulated, *ex ante*, there is often little direct evidence to hand; by way of contrast, after the fact, *ex post*, there is much empirical data and many media and scholars interested in testing matters. The challenge, therefore, is to introduce mechanisms or processes that enable more reliable predictions of likely outcomes *prior* to the event or implementation (Ferreira et al., 2024).

We believe that the means to achieve this desirable goal has already been articulated some 2400 years ago by Aristotle ([350 BCE] 2015). To be precise, in *Prior Analytics* Aristotle set out one of the most radical notions in the history of ideas: that we might systematise thought to generate new knowledge. In particular, Aristotle set out the power of the syllogism to discover new truths from those already known so that ‘certain things having been supposed, something different from those supposed *results of necessity* because of their being so’ (emphasis added; Aristotle, 2015). We will define syllogism more thoroughly in the next section but for now it will be helpful to understand it as a sequence of carefully constructed statements that lead to a conclusion. The mathematician and philosopher Leibniz declared in the 17th century that the syllogism was ‘the finest, and indeed one of the most important [inventions] to have been made by the human mind’ (quoted in Lu-Adler, 2016). Furthermore, Kant asserted that nothing could be added to Aristotle with respect to the topic of logical thought. Yet most modern readers have probably never heard of the word ‘syllogism’ and are also probably unaware of its power to deduce new truths *ex ante*.

This is a rather strange state of affairs, especially considering that formal logic was part of the trivium and considered an essential component of education right up until the 1950s. Indeed, until recent years deductive proofs – a species of formal logic – were a feature of high school mathematics curriculum. However, it seems that teaching formal logic is no longer a priority – nonetheless, it is hard to conclude that the careful use of reason is no longer required (Drew, 2025).

This paper thus addresses a glaring gap in conventional scholarly exposition, in addition to providing an important remedy for public policy disappointments. Accordingly, in the next section we set out the basic ideas of the practical syllogism with an emphasis on accessibility and applicability. Thereafter, we explicate on our methodology which seeks to demonstrate the value of the practical syllogism with respect to a vexed area of real-world contemporary policy challenge. Following this we identify the apparent syllogism at the heart of the reform that we take as our exemplar and also work through the steps required to test its soundness. We conclude this essay with some thoughts around the generalisability of the practical syllogism for the devising of more efficacious public policy.

## The Theory of Practical Syllogism

The excellence of humans is to reason. This is the thing that sets us apart from every other form of life on this planet (Finnis, 1998). Reasoning involves planning, choosing, abstracting, negating and reflecting (Drew, 2025). According to Aristotle’s famous function argument when a thing performs the unique function of its type, then it is considered excellent; when it fails to perform its unique function, then it is defective. A person who habitually uses reason is virtuous and hence excellent (Rhonheimer, 2000). Public policy constructed according to reason might also be considered, by extension, to be virtuous and excellent (D’Aquino, 1949).

Practical syllogisms are a species of syllogism cast more broadly, so this is the place that we must start in our explication. A syllogism is a formal system of arriving at new truths from other probable or certain truths. When people or public policy makers correctly employ syllogistic reasoning then spurious conclusions are not possible, and excellent reasoning is guaranteed.

For reasoning to be sound, it must be both valid and true. The workhorse of the syllogism is the premise. A premise is merely a statement that makes a claim about a thing – that is, a premise is composed of a predicate about a subject. Typically, a syllogism employs two premises to deduce or infer a conclusion. Validity means that if all of the premises were in fact true, then the conclusion must be undeniable. Thus, when testing a syllogism for soundness we first assume that the premises are indeed true (even though we have not yet assessed the truth value at this point). Once the validity of a syllogism has been established, the truth value of each of the premises must then be tested. ‘True’ premises may be either absolutely true or at least highly probable. Only if both premises are valid, and also true or highly probable, is it reasonable to follow the conclusion of the syllogism (Mitchell, 1967).

The standard exemplar syllogism, employed since at least the Middle Ages, will likely illuminate matters:

Premise 1: All men (*sic*) are mortal.

Premise 2: Socrates is a man.

Conclusion: Therefore, Socrates is mortal.

If this syllogism is both valid and true then we will be led to new undeniable truth. First, we test the validity of the syllogism – if all men are indeed mortal and Socrates is a man, clearly we must accept the conclusion that Socrates is mortal. Next, we test the truth value of each premise: (i) yes, we do indeed agree that all men (and women) die at some point, so this is (at least) a highly probable statement; (ii) yes, we do have historical accounts of Socrates from Plato, Xenophon and Aristophanes that make it highly probable to believe that Socrates was a man. Thus, we must agree with the conclusion that Socrates is/was mortal.

This rather trivial example does illustrate the power of the general syllogism for arriving at necessary new knowledge. The practical syllogism has a slightly different purpose and a slightly different form. The practical syllogism is a formulaic way to conduct good reasoning directed to action (Rhonheimer, 2000). Because of this task it deals first in premises based on ends (the good outcome sought) and then on means (the apparent way to achieve the said good outcome). The practical syllogism results in a conclusion that is a call to action. Thus, the practical syllogism has the following basic structure:

Premise 1: the good end (outcome) sought (universal), sometimes expressed as an imperative.

Premise 2: the means (apparent way) orientated towards the end (particular).

Conclusion: a command to action.

Note that the first premise is a universal claim – that is, it is a claim that is not context specific. The second premise is particular (i.e. context specific) and the conclusion is also particular.

An example of a practical syllogism, adapted from Aristotle’s (1998) *Nicomachean Ethic*, will prove illuminating:

Premise 1: People should eat healthily.

Premise 2: Dry food is healthy.

Conclusion: Therefore, I should eat dry food.

Premise 1 is the universal end (health); premise 2 is the particular means (dry food); and the conclusion is a call to action.

It is also necessary to understand the conventions for naming terms (and hence some of the premises). There are three terms in a syllogism: the major, the minor and the middle. The major term is identified as the predicate of the conclusion (‘to eat’, in the example above); the minor is the subject in the conclusion (‘dry food’ in the illustration above) and the middle term is the thing that is common to both premises (‘health/ily’). There must be three and only three terms. Moreover, the middle term proves to be crucial because this is the thing that connects the two statements and allows for sound conclusion making. In formal logic, the premises take their names from the terms that they contain – thus above, premise 1 is the major premise and premise 2 is the minor (see, e.g. Mitchell, 1967).

The relevance of the practical syllogism for sound public policymaking is clearly evident. If we construct a valid syllogism, and each premise is true, then the outcome is, by necessity, effective public policy. Otherwise stated, the thus far neglected syllogism is clearly a tool that might be applied to radically reduce the numbers of public policy failures.

Moreover, the syllogism also potentially holds the key for better public policy. Drew (2025) illustrates the utility of the practical syllogism in *Creating Human Value*. He notes that each premise is an important and necessary step for efficacious policy. In the first premise (the major term), we set out what we think constitutes a universal good end. At this step the ultimate objective is laid bare, and people can clearly assess whether the proposed outcome would be considered a good policy result – if we agree that this is so, then we are all on the same page; if not, then there might seem little point in proceeding further. Moreover, it is important for all involved in policy – both the policymakers and the policy subjects – to understand what the aim of the exercise is. One might assume that policy objectives are always clear, but in practice they often aren’t (Drew, 2025). In the second premise, we set out a particular proposed means for achieving the objective that we have agreed is worthy of striving for. This is also an important step because it is at this point that we test (i) whether the proposed means does indeed lead to the nominated desired end, and perhaps even more crucially, (ii) if the means does lead to the end, whether it is the *best* means for achieving the desired outcome. This is where evidence and debate around alternate means might be profitably directed. The conclusion is a clear statement of the policy action that should proceed to be operationalised.

To demonstrate the importance of the practical syllogism, we will later spend some time in setting out a real-world public policy example. As will be seen at this time, the failure to clearly specify ends and means can result in failed policy and much unintended harm. Before proceeding along this course, however, we must first briefly consider two main objections that have been raised with respect to the practical syllogism. Readers might note that philosophers are trained to find potential problems and caveats to all manner of things – therefore, the existence of objections is not particularly remarkable. Moreover, Flannery (2009) and others have expertly refuted the supposed problems.

The first potential objection is that public policy makers might construct a practical syllogism but fail to act according to the resultant command.^1^ However, doing so would be completely unreasonable and also completely at odds with the first self-evident precept of natural law (the philosophical ‘home’ of the practical syllogism; Finnis, 1998). People do, in fact, fail to act reasonably on all manner of things but we don’t usually declare the (universal) thing or technique wrong simply because a particular person chooses to respond to it in an unreasonable way.

The second objection is a species of the first: it occurs when a person rejects the particular means of a practical syllogism and pursues a different means to achieve the same stated end. This objection highlights the defeasibility of means – a supposed weakness that Drew (2025) has indeed shown, to be a great strength when it comes to public policy formulation. Means can typically be defeated by better options – for instance, I might find that contra Aristotle, broccoli will yield better outcomes for health more quickly. But this does not mean that following our original command (to eat dry food) was in some way wrong – merely that it was not the best means to the end of health. Indeed, if explicitly stating the second premise results in debates that yield alternative means that are later shown to be more efficacious, then clearly the process will have been to the overall betterment of policy development. Otherwise stated, thinking through and debating different paths to an agreed good end encourages policymakers to canvass and test alternatives with a view to properly surveying the policy possibility frontier and selecting the best option.

In the section that follows, we will set out a methodology to demonstrate the full utility of the approach that we have advocated with respect to a real-world contentious matter.

## Methodology and Context

Thus, far we have illustrated the power of the syllogism with respect to standard exemplars. However, we still need to illustrate the power of the practical syllogism for making better public policy. Otherwise stated, we need to demonstrate the potential of the practical syllogism for better *ex ante* decision-making that might result in less *ex post* disappointments.

To do so, it would seem helpful to employ a well-documented contentious public policy that is also capable of *ex post* empirical assessment. Documentation is important so that a practical syllogism can be constructed that would have been available to public policy architects and decision-makers *ex ante*. Contention is useful because it suggests either (i) disagreement about universal ends, (ii) disputes about particular means, or (iii) a failure to clearly articulate the aforementioned. In all cases, discernment of the apparent practical syllogism should lead to important insights. The ability to evaluate the public policy intervention with *ex post* empirical data is important so that we can test the veracity of our claims based on an earlier analysis of the apparent syllogism.

The methodology used for the *ex ante* assessment is to first derive an apparent practical syllogism based on source documents that were publicly available prior to the execution of the policy, according to the rules of formal logic outlined in the earlier section. Once this has been done, the soundness of the reasoning can be assessed. To do so we must evaluate the validity of the syllogism, and after doing so the truth value of each premise. Once this has all been done, we can form necessary conclusions based on the form of the logic employed by public policy architects. Notably, according to Aristotle (2015) nothing else is required to know whether the call to action was indeed sound and hence worthy of execution.

Nevertheless, readers are likely to wonder whether our evaluation of the policy – on the basis of *ex ante* logic alone – was indeed concordant with what transpired in the ‘real world’. Accordingly, in the second stage of the analysis we conduct an *ex post* empirical evaluation using sophisticated econometrics which yield evidence with statistical certainty. Before describing the procedures that we employ, however, we must first outline the context for our study.

Our study is based on the 2016 forced amalgamation of local governments in the largest state of Australia (with respect to both population and economic output), New South Wales (NSW). This is an ideal context for our work because the process was extremely well documented and also very contentious (resulting in protests, court actions and the political downfall of the state government reformers), and sufficient time has now elapse to evaluate outcomes. In addition, the last minute reprieve of twenty-six local governments due to Court actions means that we have a cohort of (escapee) local governments that form a reliable comparator group upon which to draw statistical inference (typically scholars compare outcomes to the entire remaining cohort of local governments but this practice likely introduces bias given that local governments selected for amalgamation generally have different characteristics from those which are not). The escapee and amalgamated cohorts are not precisely the same here, but this is not what is required anyhow (see below regarding the assumption of the empirical technique used). What is key in our confirmatory empirical work is for it to be reasonable to believe that performance of the amalgamated cohort might have been expected to be at least similar to the trend in actual performance of the escapee cohort. Given that each cohort had statistically similar pre-amalgamation performance, and that each was originally selected for treatment according to the same criteria by the same policy architects, then this assumption seems reasonable.

Local government in NSW is now composed of 128 entities that fulfil a comparatively limited remit focused on roads, rubbish and recreation (McQuestin et al., 2020). In addition, around two-thirds of the local governments also provide water and sewer services and this typically accounts for around a quarter of expenditure when present. Thus, most services offered by local governments in NSW are orientated towards properties notwithstanding emerging forays into home care, childcare and health by some entities. The single largest area of expenditure are roads which is reflective of the fact that local governments are responsible for over eighty-five percent of the nation’s thoroughfares (Drew et al., 2022). The funding for local government is derived almost equally in aggregate from unimproved land-based taxes, fees and intergovernmental grants, respectively.

To conduct an *ex post* assessment of the amalgamation outcomes – and thus to confirm the veracity of our *ex ante* evaluation – we conducted difference-in-difference (DiD) regression on a wide and long panel of data (eight years before and six years after).

**Figure 1.**
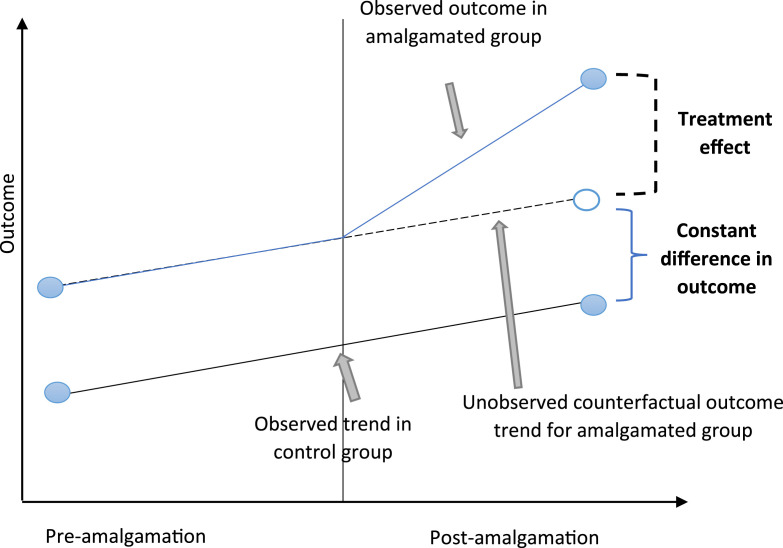
Difference-in-difference regression concepts.

DiD tests the effect of treatment (in this case, the eighteen amalgamations) by isolating the outcomes for the treated cohort after the event with reference to a counterfactual trend established from a reasonable comparator group (in this case the twenty-six escapee local governments) (see Figure 1) (Reingewertz, 2012). Like all econometric exercises, controls are put into place to allow for *ceteris paribus* judgements. DiD is widely considered to be the most robust technique for empirically assessing post-policy treatment effects,^2^ and we remind readers that the exercise is purely conducted as an assurance to the main analysis (construction and critique of the *ex ante* practical syllogism).

The main variable of interest to us is the effect of treatment on the unit costs of amalgamated local governments (operational expenditure per property.^3^) As will be seen, a key objective of the public policy revolved around reducing these costs. The specification for the DiD is as follows:

$$E_{i,t}=\beta_{0}+\beta_{1}{period}_{i,t}+\beta_{2}{treated}_{i,t}+\delta {period}_{i,t}\cdot {treated}_{i,t}+\gamma X_{i,t}+\varepsilon_{i,t}$$

where **E** is the operational expenditure per property assessment for each local government *i* in the financial year *t* (as per the subscripts), $\beta_{0}$ is a constant term, $\beta_{1}$ and $\beta_{2}$ are slope parameters for the variables that follow them, **period** is an indicator variable assigned the value of one in the years following the amalgamations, **treated** is an indicator variable given a value of one for the local governments which were amalgamated, δ is the variable of principal interest (DiD estimator reported later in our results section), **X** is a vector of local government controls and ε is the independent and identically distributed error term. Data was transformed by the natural log, as is the usual econometric practice, where important skewing was indicated by diagnostic tests. Table 1 lists the variables and their definitions employed. It might be noted that this is the standard set of regressors used in all Australian econometric work (see Drew et al., 2022; McQuestin et al., 2020 for standard justifications for each variable employed).

**Table 1.** Variables Employed in the *Ex Post* Empirical Investigation.

Variables	Definition	Mean	Standard deviation
Operational expenditure per assessment (logged)	Staff, material and contracts, plus other expenditure per property assessment ($000)	1.09	0.34
Total property assessments (logged)	Total number of properties in the local government area	9.83	1.17
Population density (logged)	Number of residents divided by the local government area	4.60	3.42
Under 15	Percentage of residents under 15 years	18.21	2.32
Aged (logged)	Percentage of residents receiving the aged pension	2.27	0.43
Disability support pension (logged)	Percentage of residents receiving a disability support pension	0.98	0.65
Newstart Allowance (logged)	Unemployment welfare allowance	0.74	0.61
Aboriginal and Torres Strait Islander percentage (logged)	Percentage of Aboriginal and Torres Strait Islander residents	0.47	1.28
Non-English-speaking background (logged)	Percentage of residents from a non-English-speaking background	2.14	1.23
Median employee income (logged)	Median employee income of residents	10.75	0.46
Sealed roads (logged)	Length of sealed roads (kms)	5.96	0.82
Unsealed roads	Length of unsealed roads (kms)	419	575
Total federal grants (ln)	Financial assistance grants received ($), per assessment	5.38	1.05
Water/Sewer	Indicator variable where the conduct of a water or sewer business = 1, otherwise 0	0.45	0.50

Thus, with methodologies in hand for both *ex ante* and *ex post* evaluations we are now ready to demonstrate the power of the practical syllogism for better public policymaking. This is the task to which we now turn our attention.

## Results and Discussion

In this section, we will show that practical syllogisms have great utility for discerning necessary truths *ex ante*. We will then assure the veracity of our formal logic deductions in the *ex post* evidence that follows.

### A Syllogistic Ex Ante Evaluation of the NSW Amalgamations

We must first establish the apparent syllogism in operation prior to the execution of the amalgamations before we can test whether the reasoning was indeed sound. Some scholars contend that the basis for policy reasoning ought to be stated clearly by proponents from the outset (see Drew, 2025); however, it is sadly often not the case. We believe that people who lived through the NSW amalgamation reform would be in no doubt of the desired end and proposed means for achieving the objective (see, e.g. Abelson & Joyeux, 2015). However, we nevertheless set out a small subsample of the compelling evidence to substantiate the formulation of an apparent operative practical syllogism.

As we have seen, the major premise is a statement of the desired end, which has a universal nature. The program under investigation was originally named *Destination 2036* and its steering committee noted that:

Financial sustainability is arguably the key requirement to achieving strong and sustainable councils that can deliver services that the community wants and can afford. Financial sustainability was identified as the most important challenge currently facing councils in NSW (Destination 2036 Steering Committee, 2012).

The reins of the policy reform were then passed on to the Independent Local Government Review Panel (ILGRP, 2012) who titled its first report *Better, Stronger, Local Government: The Case for Sustainable Change*. The key piece of evidence relied upon by the ILGRP (2012, 2013a, 2013b) was an analysis of the *Financial Sustainability of the New South Wales Local Government Sector* by the NSW Treasury Corporation (TCorp) dated March 2013. ‘Following a three-year independent review of local government, the NSW Government [then] released its *Fit for the Future* (FFTF) program in September 2014’ (LGNSW, 2014). A sustainability evaluation was subsequently conducted by the Independent Pricing and Regulatory Tribunal^4^ (IPART, 2015). This led to the NSW state government – which ultimately forced the amalgamations – to issue a media release that headlined ‘Report Card Shows Majority of Councils Still Operating in the Red’ (Toole, 2015). Forced amalgamations transpired shortly thereafter.

It is thus clear that financial sustainability was the main motivation and focus of activity associated with the reform. We therefore infer the universal end of the practical syllogism to be:

Major Premise: Local Government ought to be financially sustainable.

The minor premise is a statement of the particular means posited to achieve the aforementioned universal end. The chosen means is somewhat obvious given how the reform ended – amalgamations – but should nonetheless be supported with evidence from the time before the May 2016 executions given that this is a demonstration of *ex ante* logic.

From its very first report the ILGRP (2012) frequently hinted at the principal means that it would ultimately recommend, thus we read:

To be an effective partner in the broader system of government, local government must be both truly ‘local’ in the way that it relates to communities, and have the ability to address problems and emerging issues at a larger scale (emphasis added; ILGRP, 2012: 10).

This expression of latent reasoning was also evident in later reports:

It is also clear that the financial base of the sector is in urgent need of repair: many councils face very serious problems that threaten their sustainability and provision of adequate services to local communities. Put simply, there are too many councils chasing too few resources (ILGRP, 2013a: 6).

Taxpayers should not be expected to increase grant funding indefinitely to support councils that are unnecessarily small, lack capacity, and build excessive costs into the system (ILGRP, 2013b: 72).

In its final report, the ILGRP (2013b) also set out ‘merger and boundary change options’ (p. 104) that employed the frequent refrain that a given local government was ‘too small to warrant a separate entity’ (see, e.g. page 91).

Ultimately, the IPART (2015: 2) evaluated the ensuing ‘fitness’ proposals and recommended amalgamations based on the following assertion:

To be assessed as fit, councils must have demonstrated they have sufficient scale and capacity^5^ and are financially sustainable (IPART, 2015: 2).

It is thus abundantly clear^6^ that amalgamation was the proposed particular means for achieving the earlier stated universal end. We therefore infer the minor premise of the apparent practical syllogism to be:

Minor Premise: Larger local governments would be more sustainable.

We note that this reconstruction of an apparent operative practical syllogism is entirely in keeping with what did actually happen – concerns were raised about financial sustainability and local governments were subsequently amalgamated in May 2016. The conclusion of the apparent practical syllogism is thus entirely self-evident:

Conclusion: Therefore, we must make local government larger to attain greater sustainability.

To summarise, the apparent *ex ante* practical syllogism was:

Major Premise: Local government ought to be financially sustainable.

Minor Premise: Larger local governments would be more sustainable.

Conclusion: Therefore, we must make local government larger to attain greater sustainability.

Now that we have derived the most plausible apparent syllogism, we are in a position to test it according to the rules of formal logic. To be a fair test, we should only draw on data available to decision-makers *ex ante*.

To be sound reasoning, a syllogism must be both valid and the premises also true (or at least probable). To test validity, we first ask ourselves whether the conclusion would be undeniable if all premises were indeed true. Thus, if we believed that local governments should be sustainable, and if larger local governments would definitely be more sustainable, then it would seem undeniable that we must make local governments larger (clearly amalgamation). The syllogism is therefore valid.

The second criteria relates to the truth value of the premises. That local government ought to be financially sustainable seems self-evident. Unsustainable local governments ultimately collapse and can no longer perform their functions (Aristotle’s function argument): it is thus hard to believe that anyone might try to assert that the major premise was untrue. The minor premise, however, may be a very different matter.

As we have noted, the key piece of evidence used by the ILGRP, IPART and the NSW Government was the assessment of financial sustainability conducted by TCorp in 2013. This report provided a rating for each local government area according to the following categorisation listed in descending order of strength (numbers of local governments achieving a particular assessment in parentheses):

Very Strong (0).

Strong (2).

Sound (32).

Moderate (79).

Weak (34).

Very Weak (5).

Distressed (0).

**Figure 2.**
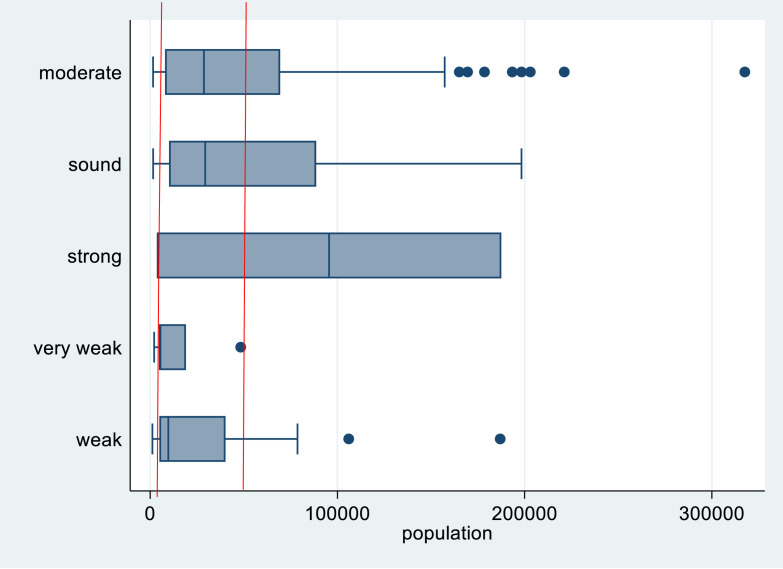
Financial sustainability ratings and local government population size.

The Australian Bureau of Statistics lists populations for each local government area, and these were also available to decision-makers in (2013).^7^ We therefore conducted some rudimentary statistical tests to ascertain the truth value of the minor premise. The first is a boxplot of financial sustainability ratings and population (Figure 2):

We have added in some red lines so that readers can quickly perceive the range of population values that were common to all ratings. This was a relatively large domain of 3517 through to 48,348. Otherwise stated there were very weak local governments with a population of just 3517 but there were also strong, moderate and sound ones at this size also. Indeed, an astounding 103 local governments had a population in the domain occupied by all five financial sustainability ratings. Given this considerable overlap for most ratings, it would not be sensible to conclude a firm association between size and sustainability (this is particularly evident in the three most common assigned ratings).

To confirm matters we also conducted an analysis of variance – once again, a pretty rudimentary task that should have been well within the capacity of decision-makers at the time. In Table 2, we detail the (Bonferroni) correlation matrix for the various putative associations (note Bartlett’s test for equal variance Prob > chi^2^ = 0.800 and log transformations have been applied as appropriate) (Table 2).

**Table 2.** Bonferroni Correlation Matrix ANOVA.

	Moderate	Sound	Strong	Very weak
Sound	0.038 (1.000)			
Strong	0.016 (1.000)	−0.023 (1.000)		
Very weak	−1.069 (0.899)	−1.107 (0.920)	−1.085 (1.000)	
Weak	−0.642 (0.225)	−0.681 (0.435)	−0.658 (1.000)	0.426 (1.000)

This ANOVA also fails to provide any evidence in support of the critical minor premise. In fact, the evidence available at the time casts significant doubt on the particular means statement.

Given that the minor premises seem to have been far less than probable,^8^ the rules of formal logic suggest that the apparent practical syllogism was not sound reasoning. Thus, according to the form first set out by Aristotle (2015) we have ‘from certain things being supposed [especially regarding the unlikely nature of the minor premise], something different from those supposed results of necessity [that is, an unsound call to action]’. Otherwise stated, by using the finest invention of the human mind – to paraphrase Leibniz – we have been able to assess with certainty the outcomes of a public policy not yet executed in 2015.

Thus, if the architects of the contentious public policy under consideration had followed the process of stating and testing their practical syllogism prior to the execution of the amalgamations then things might have been very different.

### An Ex Post Evaluation of the NSW Amalgamations

To provide some additional assurance around our main analysis – the practical syllogism – we also conducted a DiD regression (see Table 3).

**Table 3.** Difference-In-Difference Regression of Amalgamation Outcomes, NSW.

Variable	Coefficient
DID estimator	0.059* (0.027)
Assessments (logged)	0.535*** (0.132)
Assessments squared (logged)	−0.011+ (0.006)
Water-sewer dummy	0.241*** (0.030)
Control variables	Yes
Number of observations	640
Coefficient of determination	0.754

*Note.* +10% significance; * 5% significance; **1% significance; *** 0.1% significance.

As can be seen, the treatment effect of amalgamation resulted in an additional 5.94% being added to unit costs, statistically significant at the 5% level, *ceteris paribus.*^9^ Reducing technical efficiency cannot possibly result in an increase to financial sustainability and runs counter to all of the opinions and undertakings made prior to May2016^10^ (see Drew et al., 2022). Even more assurance of our practical syllogism analysis can be had by contemplating the fact that one of the amalgamated local governments was placed into administration after running out of unrestricted cash (see McQuestin et al., 2020): a number of amalgamated local governments were also forced to impose hefty increases to the taxes levied on their residents, including Cootamundra-Gundagai (53.5%), Snowy Valleys (35.95%), Canterbury-Bankstown (36.34%), Federation (39.2%) and Armidale (58.8%) (IPART, 2024). Further assurance can be found in other scholarly work that has empirically demonstrated that the policy failed to achieve its objectives (see, for examples, Drew et al., 2022; McQuestin et al., 2020). It therefore seems pretty certain that the larger local governments did not attain greater sustainability as the public policy architects had hoped might be the case. Combined, these results provide a high level of assurance around the veracity of our earlier main analysis – the construction and critique of the practical syllogism.

Thus, there can be little doubt that had policy architects employed the practical syllogism *ex ante*, that much disappointment might have been averted *ex post*.

## Conclusion: Is It Time to Return to Syllogistic Reasoning?

Our exposition has clearly demonstrated that – to paraphrase Leibniz – the finest and most important invention of the human mind has much to offer public policy architects. Specifically, it is now beyond doubt that careful articulation and testing of the practical syllogism would have exposed logical flaws and thus averted yet another unnecessary public policy failure (Tavares, 2018). It is notable that scholars have never before established the link between public policy failures and formal logic failures. We have thus uncovered a powerful – albeit relatively simple – remedy that might, in time, put an end to the exasperation of scholars regarding avoidable policy failures (see, e.g. Gendzwil et al., 2021).

There are also a number of other practical benefits that might be gained beyond simply attaining the laudable goal of averting more policy mistakes. For instance, the practical syllogism also seems ideal for quickly communicating policy to the citizenry. It succinctly sets out the desired outcome and means for achieving same in a way that ought to efficiently convey what government is doing and why. If more people were aware of what the government was seeking to do then this might be expected to result in more co-operation, which could only be a good thing for the policy itself, and society more broadly (Booth, 1974).

In addition, studious application of the practical syllogism ought also to lead to improved public policy outcomes. Even had amalgamation been a sound call to action, it might still have been the case that alternatives such as removing taxation limitations, or increasing grants, could have produced even better results. By clearly articulating the proposed means for a desired end – and then opening this up for debate and testing – it is likely that even effective policy could be further improved upon. Thus, the syllogism not only holds the potential to avert policy failures but also to move more of the grey area outcomes into unambiguous successes (Howlett, 2012).

It is also easy to see how our work might be generalised to different contexts and different jurisdictions. Reasoning is the excellence of humans, and the syllogism is a tried and tested formula for reliably producing sound reasoning. Thus, any time that people need to make important policy decisions is an opportunity to improve outcomes by recourse to the practical syllogism. Whether the policy refers to health or education, is being formulated in Australia or Austria, good reasoning is undoubtedly the surest path to desirable outcomes. It is hard to conceive of a single instance where it is not important to know what outcome is desired, and the best means for achieving same.

Future research should be directed to confirming our findings by constructing and critiquing practical syllogisms for other well-documented and contentious public policies. Ideally, this would take place with respect to different sectors in both Australia and abroad. It is always possible that happenstance might occasionally produce outcomes contrary to good reason, especially if the minor premise is probable rather than certain. However, we would expect the bulk of careful studies to concord with our own work because it is reasonable to expect good public policy outcomes from good reasoning. Further, as more evidence emerges to support the power of practical syllogisms, we anticipate that this will translate into a greater awareness and use by practitioners, and hence better policy outcomes.

In sum, many may have forgotten the syllogism of Aristotle, but the need for good reasoning persists nonetheless – perhaps it is thus time for public policy architects to turn their minds to studiously deploying a radical notion formalised some 2400 years ago.

## Notes

1. In Book 7 of the *Nicomachean Ethics,* Aristotle (1998) appears to refute this idea by (i) concurring with Socrates that people should not act contrary to their knowledge of good and (ii) noting the importance of the particularity of the minor premise (which ‘encourages’ the person to respond to the syllogism provided that they are not being restrained in some way).

2. The major alternative to DiD would seem to be to conduct some crude comparison of the post-amalgamation changes to operating expenditure. However, an approach such as this – sans controls for changes to demographics and other important determinants – would have no reference to what might have been (the counterfactual) and thus offer little in the way of robust statistical judgement.

3. As we noted before, most Australian local government services are still directed to properties: it is therefore the most reasonable numeraire. However, we also re-ran the DiD with population and found similar results (available from the corresponding author).

4. This was a confirmatory report based on a subset of the TCorp (2013) financial ratios, a new contentious efficiency ratio (see Drew & Dollery, 2015), as well as the population thresholds established by the ILGRP (2013b).

5. A population threshold largely drawn from the aforementioned ILGRP (2013b) report.

6. Later the Chair of the ILGRP tried to argue that amalgamation was never his intended means for achieving the end of financial sustainability – a claim that was met with some incredulity by scholars. For instance, Abelson and Joyeux (2015: 351) observed that ‘this assertion may surprise readers of the ILGRP report, which appears at many points to link the case for larger councils with concerns about financial sustainability (see, e.g. pp. 3, 7, 18, 26, 72–73 and 98)’ (Abelson & Joyeux, 2015, p. 351).

7. Population was the erroneous unit employed by decision-makers. It is therefore the unit that we employ for our assessment of the minor premise, in keeping with our remit.

8. There was also a number of scholarly papers available online before the execution of amalgamations that cast significant doubt on the logic of the reform. These included Drew and Dollery (2015), Drew and Dollery (2014), Abelson and Joyeux (2015) and Drew et al. (2015).

9. Relevant diagnostic tests indicated that the residuals of the model were normally distributed at the 5% significance level (*p* = .2412) and that the two cohorts of councils (those which were amalgamated and those which escaped amalgamation) followed similar pre-treatment trends (*p* = .6284). Logarithmic transformations were applied to variables with skewed distributions.

10. The media release spruiking the amalgamations told residents that their local governments would be more efficient and hence stronger – see Baird (2015).
